# Association of healthy eating index and anthropometric indices among primary school girls in southeast of Iran: a cross-sectional study

**DOI:** 10.1186/s40795-024-00847-9

**Published:** 2024-02-27

**Authors:** Nooshin Jannati, Mohammad Reza Mahmoodi, Leila Azadbakht

**Affiliations:** 1 Department of Community Nutrition, School of Nutritional Sciences and Dietetics, Tehran University of Medical Sciences (TUMS), Tehran, Iran; 2 Physiology Research Center, Institute of Neuropharmacology; Department of Nutrition, School of Public Health, Kerman University of Medical Sciences, Kerman, Iran

**Keywords:** Anthropometric indices, Healthy eating index, Kerman, Overweight, Obesity, Thinness

## Abstract

**Background:**

The school-age period is marked by substantial advancements in physical and cognitive development, highlighting the importance of assessing the diet quality and its impact on body weight and height. The main objective of this study was to evaluate the association between diet quality and selected anthropometric indices of primary school girls in southeast of Iran using the healthy eating index-2015 (HEI-2015).

**Methods:**

This cross-sectional study involved 330 students aged 6–12 years from 10 primary schools in Kerman City. Standard protocols and a dish-based food frequency questionnaire were employed to evaluate anthropometric indices and dietary intake. The HEI-2015 was utilized to assess the quality of participants’ diets, with a total score ranging from zero to 100, based on thirteen food score components.

**Results:**

In the present study, older participants had higher HEI scores (*p* = 0.02). Additionally, participants in the highest tertile of HEI score had greaterodds of being overweight (OR: 2.13; CI = 1.17–3.85, *P* = 0.011) and had higher intakes of whole fruits, total fruits including fruit juice, whole grains, total protein foods, seafood and plant proteins, greens, and beans (*p* < 0.05). However, no significant association was found between HEI score and other anthropometric indices, obesity, and thinness.

**Conclusions:**

The study found no significant association between HEI scores and the likelihood of being thin or obese. However, children with the highest HEI scores were more likely to beoverweight. Therefore, it is recommended to implement health programs for primary school girls in Kerman to improve their eating habits and reduce the risk of overweight and obesity.

## Background

Childhood is a critical period for the development of the mind and body. Assessing child growth is crucial for community health and nutritional well-being [1]. Approxiamtely 33% of children worldwide suffer from malnutrition [2]. In Iran, the prevalence of malnutrition, as measured by weight-for-age, height-for-age, and weight-for-height indices, was found to be 46.6%, 36.9%, and 53.3%, respectively [3]. Malnutrition in children can lead to growth failure, death, and disease due to inadequate or unsuitable food consumption [4, 5]. Therefore, evaluating children’s dietary intake is essential.

Assessing the quality of dietary intake, rather than just quantity, is important for a comprehensive evaluation of an individual’s food and beverage consumption. Poor diet quality can significantly affect mental and physical well-being, leading to conditions such as stunting, obesity, and cardiovascular disease [6]. Diet quality assessment is complex, as determining the quality based on just one nutrient or a single day’s food consumption is not a reliable indicator of the overall diet quality.

Various dietary indices have been developed to assess diet quality. The healthy eating index (HEI) is designed to evaluate diet quality by comparing it to the recommendations of Dietary Guidelines for Americans [7, 8]. It comprises 13 components representing different food groups for individuals aged two years and older and can predict the risk of various health consequences [9].

Recent evidence shows that food insecurity rates among healthy individuals in Iran are higher than the global average [10]. Those who experience food insecurity tend to have lower diet quality compared to those who do not face food insecurity [11].Additionally, Kerman, a province in southeast of Iran,is among the seven provinces facing food insecurity [12].Therefore, evaluating the diet quality of children is of great importance. The current study aimed to determine the overall diet quality using HEI scores and investigate the association between HEI scores and anthropometric measures among female students in Kerman. This research is crucial for understanding and addressing the dietary needs of children in this region.

## Method

### Study design and population

This cross-sectional study collected data from 330 female primary school students aged 6 to 12, from ten girls-only schools in Kerman, Iran, between January and March 2022. Data were collected by a trained nutritionist. Quality control procedures, including regular checks for data completeness and accuracy, were implemented to minimize errors in data collection. Kerman, the capital of Kerman province, has a population of approximately 3 million. The necessary sample size was determined using the mean and standard deviation of BMI from a previous study on Iranian children aged 7–11 (Mean (± SD) = 16.0 ± 2.9 kg/m²) [13]. Using the formula: n= [(z1 -α⁄2) ² ×s²] ⁄ d² with d = 2% and alpha = 0.05, a sample size of 323 was calculated and subsequently increased to 330 for added reliability. The study was designed with 80% power. We used the cluster random sampling method to select the cases. The study protocol received approval from the Ethical Committee of the Tehran University of Medical Sciences (IR.TUMS.MEDICINE.REC.1400.603). All methods were carried out in accordance with relevant guidelines and regulations or declaration of Helsinki.

### Inclusion and exclusion criteria

The present study recruited participants based on the following inclusion criteria:

(1) Voluntary participation, as indicated by the completion of a consent form, (2) Age eligibility within 6 to 12 years, (3) No chronic diseases such as diabetes, congenital metabolic disorders including maple syrup urine disease, phenylketonuria, thyroid gland anomalies, epilepsy, and asthma, and (4) Absence of corticosteroids, thyroid medications, diabetes medications, epilepsy medications, or allergy medications usage.

The study sample was limited to exclude children whose parents refrained from completing the informed consent.

### Assessment of dietary intake

A dish-based food-frequency questionnaire (FFQ) designed for the Kerman population, along with standardized serving portions, was used to assess dietary consumption patterns over a year. This assessment was conducted by the participant’s parents. The FFQ comprised 185 individual food items. The validity and reliability of this FFQ are reported in section “Reliability and reproducibility of food frequency questionnaire”. Under standardized methodology, the frequency and portion size of all food items were converted into grams per day (g/day). NUTRITIONIST-IV software, adapted for Iranian food composition, quantified energy and nutrient content.

### Healthy eating index 2015

Evaluating of the diet quality of participants was conducted using the HEI scores. The HEI comprises 13 components divided into two subgroups: adequacy and moderation. Nine factors for nutritional adequacy included consumption of fruits (total and whole), vegetables (greens and beans), whole grains, dairy products, protein-rich foods (animal and vegetable-based), seafood, and healthy fatty acids. Moderation elements included processed grains, salt, added sugars, and saturated fats. In terms of adequacy components, the highest possible score for various food groups, such as fruits (both the total and whole), vegetables (both total and greens/beans), and protein (both total and subdivided categories of seafood and plant-based), was five points. Non-consumption of any of these food items resulted in a score of zero.

Similarly, a score of up to 10 points was attainable for whole grains, dairy, and fatty acids. The score for consuming fatty acids (consisting of polyunsaturated and monounsaturated fatty acids compared to saturated fatty acids) is zero if not consumed. A rating of 10 was assigned to moderation elements when refined grains, sodium, added sugars, and saturated fats were consumed within the recommended limits, while reaching the upper consumption limit was attributed a score of zero. The overall HEI-2015 score was determined by adding scores of these components, resulting in a score range of zero to 100 [9].

### Anthropometric measurements

Anthropometric measurements comprising height, body weight, and mid-arm circumference were measured for all children. Measurements are carried out to ensure reliability and validity by using standardized protocols and tools (CDC protocol attached as supplementary file). The person who did the measurements underwent training to ensure consistency in measurement techniques. Height was measured following established protocols, utilizing a stationary measuring instrument and positioned against a wall, with measurements taken to the nearest 0.1 cm. Participants’ body weight was assessed through a digital scale providing a precision of 0.1 kg. Participants wore minimal clothing without shoes during their body weight measurement. We evaluated mid-arm circumference via the precise measurement of the circumferential extent at the midpoint between the elbow and the shoulder, with a degree of precision of 0.1 mm. The body mass index (BMI) was computed through the division of the individual’s body weight in kilograms (kg) by the square of their height in meters (m), represented as kg/m^2^.

The criteria established by the World Health Organization [20] applied for calculating z-scores for BMI-for-age (BAZ), height-for-age (HAZ), and weight-for-age (WAZ). BAZ were classified according to their weight status, which included obese (BAZ ≥ 2 SD), overweight (1 SD ≤ BAZ < 2 SD), thin (− 3 SD < BAZ ≤ − 2 SD), and severely thin (BAZ ≤ − 3 SD). HAZ categories can be classified as stunted (− 3 SD < HAZ ≤ − 2 SD) and severely stunted (HAZ ≤ − 3 SD) [14].

### Demographic and socio-economic status

A valid and reliable questionnaire was applied to evaluate demographic and socioeconomic status (SES) among the Iranian participants to examine correlation between SES and health outcomes [15]. Questions of the education and employment status of parents, household size, property ownership or rental status, possession of vehicles including the number of cars, the number of bedrooms in the dwelling, and ownership of various appliances such as washing machine, dishwasher, LCD TV, side-by-side refrigerator, air conditioner, vacuum cleaner, computer, laptop, and advanced heating system were incorporated into the survey instrument [15]. The scoring method was employed to evaluate socioeconomic status. Finally, the codes were summated and classified into three categories based on the qualitative description: weak, moderate, and rich.

Supplement intake and age of participants were recorded in a general questionnaire. The short form of the International Physical Activity Questionnaire (IPAQ) evaluated participants’ physical activity (PA) [16]. The scores were computed based on the frequency and duration of light, moderate, and vigorous physical activities and sedentary episodes over the preceding seven-day period. The quantification of PA is typically conveyed through the expression of metabolic equivalent hours per week. The PA level stratified according to three categories: low physical activity, defined as less than 600 METs-minutes per week; moderate physical activity, ranging between 600 and 3,000 METs-minutes per week; and high physical activity, exceeding 3,000 METs-minutes per week [17].

### Reliability and reproducibility of food frequency questionnaire

In this study, we assessed the reproducibility of the questionnaire by having 56 parents complete it twice, 12 weeks apart. The intra-class correlation coefficient was applied to measure the reproducibility. Additionally, three 24-hour recalls were collected during the study to assess validity. Pearson correlations and the Wilcoxon signed-rank test evaluated the validity. The strength of the relationship for data validity was interpreted using specific correlation rating interpretations. For Pearson statistics, a correlation of 0.10 to 0.30 was considered weak, 0.30 to 0.50 was moderate, and above 0.50 was strong. An ICC value of 0.00 to 0.10 indicated virtually no relationship, 0.11 to 0.40, 0.41 to 0.60, 0.61 to 0.80, and 0.81 to 1.0 indicated a slight, fair, moderate, and substantial relationship, respectively.

### Statistical analysis

Histogram curves and Kolmogorov-Smirnov test examined the normal distribution of variables. In order to compare qualitative variables across tertiles of the Healthy Eating Index (HEI), the chi-square analysis was employed. Similarly, an analysis of variance (ANOVA) test was applied to compare quantitative variables across the HEI tertiles. An analysis of covariance (ANCOVA) evaluated the relationship between dietary consumption and anthropometric measurements across the Healthy Eating Index (HEI) tertiles. Three statistical models were applied in the study, namely: Model 1 (a crude model), Model 2 (adjusted for age and energy), and Model 3 (adjusted for age, energy, supplement use, parent smoking, physical activity, and socioeconomic status). A multiple logistic regression was applied to investigate the potential relationship between diet quality and various anthropometric indicators, including obesity, overweight, and underweight. The variance inflation factor (VIF) values for all independent variables in our regression model are below 5, indicating no significant multicollinearity concerns. All statistical analyses were conducted using the Statistical Package for the Social Sciences (SPSS) version 24. A p-value of less than 0.05 was considered significant.

## Results

### Sociodemographic characteristics of study participants

Table 1 presents the demographic characteristics of the participants. The average age of participants was 9.02 ± 1.81 years. A significant association was found between age and HEI tertile (*p* = 0.02). However, no association was observed between other demographic characteristics and HEI. The rate of supplement use was 18.2%. 91.2% of participants have a low level of physical activity.

**Table 1:** Characteristics of participants (primary school girls in Kerman) in tertiles of Healthy Eating Index-2015

Healthy Eating Index-2015 tertiles						
Variable		All *N* = 330	Tertile 1 ≤ 57 *N* = 133	Tertile 2 > 57 < 60 *N* = 69	Tertile 3 ≥60 *N* = 128	*P* value
		Quantitative variables*/ mean ± SD				
Age (year) Weight (kg) Height (cm) BMI (kg/m2) MUAC (cm) Physical activity (met/min/week)		9.02(1.81) 33.81(13.03) 136.68(13.56) 17.49(3.93) 21.97(3.72) 193.87(428.30)	8.85(1.85) 32.96(13.31) 136.10(14.21) 17.15(3.94) 21.75(3.70) 160.79(327.49)	8.74(1.72) 31.55(10.67) 134.05(12.64) 17.11(3.38) 21.36(3.51) 240.56(513.30)	9.35(1.77) 35.90(13.68) 138.69(13.15) 18.03(4.15) 22.53(3.80) 203.07(469.32)	0.028 0.051 0.059 0.132 0.075 0.435
		**Qualitative variables† /N (%)**				
Grade	1st 2nd 3rd 4th 5th 6th	17.6% 15.5% 16.7% 16.1% 17.3% 17.0%	28(8.5%) 22(6.7%) 24(7.3%) 17(5.2%) 24(7.3%) 18(5.5%)	13(3.9%) 12(3.6%) 13(3.9%) 11(3.3%) 13(3.9%) 7(2.1%)	17(5.2%) 17(5.2%) 18(5.5%) 25(7.6%) 20(6.1%) 31(9.4%)	0.233
Socioeconomic status	Low Medium High	56.4% 41.5% 2.1%	67(20.3%) 64(19.4%) 2(0.6%)	48(14.5%) 19(5.8%) 2(0.6%)	71(21.5%) 54(16.4%) 3(0.9%)	0.087
Supplement	Yes No	18.2% 81.8%	27(8.2%) 106(32.1%)	10(3.0%) 59(17.9%)	23(7.0%) 105(31.8%)	0.596
Parents smoking	Yes No	11.5% 88.5%	18(5.5%) 115(34.8%)	6(1.8%) 63(19.1%)	14(4.2%) 114(34.5%)	0.573
Physical activity	Low Medium High	91.2% 8.5% 0.3%	124(37.6%) 9(2.7%) 0(0.0%)	60(18.2%) 9(2.7%) 0(0.0%)	117(35.5%) 10(3.0%) 1(0.3%)	0.407
Father’s education	High school Diploma College	17.9% 49.1% 33.0%	25(7.6%) 64(19.4%) 44(13.3%)	13(3.9%) 31(9.4%) 25(7.6%)	21(6.4%) 67(20.3%) 40(12.1%)	0.889
Mother’s education	High school Diploma College	13.0% 53.3% 33.6%	18(5.5%) 68(20.6%) 47(14.2%)	10(3.0%) 33(10.0%) 26(7.9%)	15(4.5%) 75(22.7%) 38(11.5%)	0.639
Family member	4 or less 5 to 7 More than 7	69.1% 29.1% 1.8%	92(27.9%) 41(12.4%) 0(0.0%)	54(16.4%) 13(3.9%) 2(0.6%)	82(24.8%) 42(12.7%) 4(1.2%)	0.072
Father’s occupation	Manager Employee Worker Housewife Retired Self-employment Others	2.4% 28.2% 9.1% 0.3% 0.9% 57.3% 1.8%	1(0.3%) 38(11.5%) 7(2.1%) 0(0.0%) 1(0.3%) 82(24.8%) 4(1.2%)	1(0.3%) 23(7.0%) 10(3.0%) 0(0.0%) 0(0.0%) 33(10.0%) 2(0.6%)	6(1.8%) 32(9.7%) 13(3.9%) 1(0.3%) 2(0.6%) 74(22.4%) 0(0.0%)	0.106
Mother’s occupation	Manager Employee Worker Housewife Retired Self-employment Others	0.6% 9.4% 0.3% 80.3% 0.3% 7.3% 1.8%	0(0.0%) 12(3.6%) 1(0.3%) 104(31.5%) 0(0.0%) 13(3.9%) 3(0.9%)	1(0.3%) 8(2.4%) 0(0.0%) 54(16.4%) 0(0.0%) 4(1.2%) 2(0.6%)	1(0.3%) 11(3.3%) 0(0.0%) 107(32.4%) 1(0.3%) 7(2.1%) 1(0.3%)	0.724

*The *P* value reported for the quantitative variables was resulted from one-way ANOVA, and the numbers are reported as mean ± SD. †The *P* value for the qualitative variables was calculated by the chi-square test, and the results are based on N (%). *P* value < 0.05 shows a significant level of association

### HEI scores and dietary intake

Dietary intake of the children is shown in Table 2. Participants in the higher tertile of HEI scores had a greater intake of fiber (*P* < 0.001), PUFA (*P* = 0.012), vitamin K (*P* = 0.008), vitamin B3 (*P* = 0.014), vitamin B9 (*P* = 0.042), iron (*P* < 0.001), whole fruit (*P* < 0.001), total fruit (*P* < 0.001), whole grain (*P* < 0.001), total protein food (*P* = 0.005), seafood and plant protein (*P* < 0.001), greens and beans (*P* = 0.003) and a significantly lower intake of SFA (*P* < 0.001) and added sugar (*P* = 0.021).

**Table 2:** Dietary intake of participants (primary school girls in Kerman) in tertiles of Healthy Eating Index

Healthy Eating Index-2015 tertiles				
Variable	Tertile 1 ≤ 57 *N* = 133	Tertile 2 > 57 < 60 *N* = 69	Tertile 3 ≥60 *N* = 128	*P* value
Energy (kcal/d) Carbohydrate (g/d) Protein (g/d) Fat (g/d) fiber (g/d) Cholesterol (mg/d) SFA (g/d) MUFA (g/d) PUFA (g/d)	1887.88(725.66) 259.43(2.34) 63.17(0.62) 65.20(0.92) 13.33(0.20) 262.37(5.61) 23.34(0.53) 19.68(0.27) 13.36(0.25)	1788.00(532.27) 263.07(3.26) 62.79(0.87) 64.17(1.27) 14.32(0.28) 266.70(7.78) 21.38(0.74) 19.41(0.38) 14.41(0.35)	1830.93(589.92) 264.66(2.39) 64.02(0.64) 62.70(0.93) 14.91(0.21) 258.12(5.71) 19.76(0.54) 19.07(0.28) 14.35(0.26)	0.545 0.285 0.461 0.163 0.0001 0.664 0.0001 0.308 0.012
Vitamin A Vitamin D (µg) Vitamin E (mg) Vitamin K (mg) Thiamine (mg) Riboflavin (mg) Niacin (mg) Vitamin B5 (mg) Vitamin B6 (mg) Folic acid (µg) Vitamin B12 (µg) Vitamin C (mg)	736.73(39.82) 1.35(0.08) 11.48(0.24) 160.06(3.96) 1.61(0.02) 1.50(0.02) 16.00(0.16) 5.01(0.06) 1.46(0.03) 207.56(3.68) 3.43(0.07) 92.36(3.64)	731.64(55.29) 1.36(0.12) 11.71(0.34) 159.39(5.49) 1.57(0.03) 1.49(0.03) 15.85(0.22) 5.11(0.09) 1.48(0.04) 211.22(5.11) 3.28(0.09) 98.91(5.06)	761.90(40.56) 1.53(0.09) 11.98(0.25) 176.03(4.03) 1.66(0.02) 1.47(0.02) 16.57(0.16) 5.04(0.06) 1.53(0.03) 220.64(3.75) 3.21(0.07) 102.02(3.71)	0.872 0.298 0.365 0.008 0.061 0.658 0.014 0.674 0.324 0.042 0.085 0.172
Calcium (mg) Magnesium (mg) Potassium (mg) Zinc (mg) Fe (mg) Phosphorus (mg) Selenium (mg)	761.88(18.50) 215.90(2.64) 2754.29(36.23) 8.25(0.10) 13.05(0.15) 1068.00(14.73) 0.10(0.002)	749.45(25.69) 220.00(3.42) 2859.30(50.30) 8.12(0.14) 13.62(0.21) 1084.78(20.45) 0.10(0.003)	734.28(18.85) 220.45(2.51) 2834.49(36.90) 7.97(0.10) 14.29(0.16) 1069.82(15.01) 0.10(0.002)	0.579 0.386 0.155 0.147 0.0001 0.785 0.847
Whole fruits (cup/d) Total fruits (cup/d) Whole grains (ounce/d) Dairy (cup/d) Total protein foods (ounce/d) Seafood & plant proteins (ounce/d) Greens & beans (cup/d) Total vegetables (cup/d) Refined grains (ounce/d) Sodium (mg/d) Added sugars (g/d)	1.66(0.06) 1.82(0.07) 0.47(0.06) 1.65(0.06) 6.03(0.12) 1.78(0.05) 0.37(0.01) 2.26(0.04) 11.83(0.32) 1149.44(20.52) 22.96(1.01)	2.14(0.09) 2.34(0.09) 0.70(0.09) 1.62(0.09) 6.22(0.17) 1.84(0.07) 0.39(0.02) 2.33(0.06) 10.86(0.43) 1154.89(28.49) 21.51(1.41)	2.00(0.06) 2.22(0.07) 1.24(0.06) 1.63(0.06) 6.61(0.12) 2.10(0.05) 0.44(0.01) 2.39(0.04) 10.89(0.32) 1135.23(20.90) 18.94(1.03)	0.0001 0.0001 0.0001 0.963 0.005 0.0001 0.003 0.150 0.072 0.825 0.021

SFA = saturated fatty acids, MUFA = monounsaturated fatty acids, and PUFA = polyunsaturated fatty acids. The *P* value is reported from covariance analysis, and the results are based on mean ± SD. All of the variables are adjusted for energy intake. *P* value < 0.05 shows a significant level of association

### Association between HEI and anthropometric indices

Table 3 shows the reported mean ± SD (SE) for the association between HEI and anthropometric indices. No significant association was found between HEI score and anthropometric indices.

**Table 3:** Association between anthropometric indices and Healthy Eating Index score among primary school girls in Kerman

Healthy Eating Index-2015 tertiles						
Variable		Tertile 1 ≤ 57 *N* = 133		Tertile 2 > 57 < 60 *N* = 69	Tertile 3 ≥60 *N* = 128	*P* value
MUAC (cm)	Model 1* Model 2† Model 3†	21.75(3.70) 21.87(0.25) 21.85(0.26)	21.36(3.51) 21.74(0.35) 21.74(0.36)		22.53(3.80) 22.20(0.26) 22.22(0.26)	0.075 0.534 0.470
BAZ	Model 1* Model 2† Model 3†	0.06(1.43) 0.06(0.11) 0.05(0.11)	0.15(1.44) 0.23(0.16) 0.24(0.16)		0.32(1.48) 0.28(0.12) 0.28(0.12)	0.369 0.400 0.351
HAZ	Model 1* Model 2† Model 3†	0.66(1.31) 0.64(0.10) 0.64(0.10)	0.45(1.18) 0.47(0.14) 0.45(0.14)		0.58(1.15) 0.59(0.10) 0.61(0.10)	0.489 0.613 0.568
WAZ	Model 1* Model 2† Model 3†	0.27(1.24) 0.17(0.81) 0.10(0.83)	-1.86(16.96) -1.87(1.09) -1.90(1.12)		0.45(1.47) 0.57(0.86) 0.68(0.88)	0.200 0.188 0.182

*The model 1 was resulted from one-way ANOVA, and the numbers are reported as mean ± SD. †Model 2 and 3 was resulted from covariance analysis, and the numbers are re-ported as mean ± SE. *P* value < 0.05 shows a significant level of association. Model 1: crude. Model 2: Adjusted for age and calorie. Model 3: Adjusted for age, calorie, supplement use, parents’ smoking, physical activity and socio-economic status. MUAC: mid-upper arm circumference, BAZ: BMI-for-age z-score, HAZ: height-for-age z score, WAZ: weight-for-age z-score

### Odds ratio and 95% confidence interval for weight disorders in tertiles of HEI

The findings from the multiple logistic regression analysis are presented in Table 4. Initially, a crude model indicated that individuals in the highest tertile of HEI score were more likely to be classified as overweight compared to those in the lowest tertile (OR: 2.13; CI = 1.17–3.85, *P* = 0.011). This association remained significant after adjustment for age and energy (OR:2.08; CI = 1.13–3.84, *p* = 0.022), parent education, parent occupation, supplement use, parent smoking, physical activity, and SES (OR:1.95; CI = 0.91–4.20, *p* = 0.019).

**Table 4:** Odd ratios and 95% confidence intervals for being thin, overweight or obese among different tertiles of Healthy Eating Index

Healthy Eating Index-2015 tertiles								
Variable		Tertile 1 ≤ 57 *N* = 133	Tertile 2 > 57 < 60 *N* = 69	Tertile 3 ≥60 *N* = 128			P trend	
Thin	Model 1 Model 2 Model 3	1 1 1	1.16(0.27–5.02) 1.24(0.28–5.46) 0.884(0.11–7.09)	1.70(0.54–5.36) 1.72(0.53–5.54) 4.63(0.74–28.78)		0.354 0.310 0.271		
Overweight	Model 1 Model 2 Model 3	1 1 1	0.95(0.43–2.10) 0.96(0.43–2.15) 1.18(0.44–3.09)	2.13(1.17–3.85) 2.08(1.13–3.84) 1.95(0.91–4.20)		0.011 0.022 0.019		
Obese	Model 1 Model 2 Model 3	1 1 1	1.09(0.45–2.62) 1.13(0.46–2.75) 1.79(0.58–5.50)	0.68(0.30–1.54) 0.61(0.27–1.41) 0.74(0.25–2.18)		0.378 0.257 0.260		

Model 1: crude. Model 2: Adjusted for age and calorie. Model 3: Adjusted for age, calorie, supplement use, parents’ smoking, physical activity and socio-economic status. *P* values were calculated using logistic regression. These values are odds ratios (95% CIs) by considering tertiles of HEI as ordinal variable

### Validity and reliability of food frequency questionnaire

Table 5 represents the results concerning the reliability and reproducibility of the food frequency questionnaire. The findings indicated that the newly developed FFQ is both reliable and valid. The correlation coefficients between dietary intake estimates obtained from the FFQ and 24-hour dietary recalls were 0.52 for carbohydrates, 0.54 for proteins, and 0.51 for fat. Additionally, the Wilcoxon signed-rank test showed no significant differences between the FFQ and 3-day dietary records for most nutrients (*p* > 0.05). The intra-class correlation coefficients, used to measure the consistency of the FFQ, ranged from 0.54 to 0.77. Overall, this FFQ is considered a suitable tool for assessing dietary intake in specific age groups.

**Table 5:** Reproducibility and validation study: Correlation coefficient and Wilcoxon Signed Rank Test for validity and ICC for reproducibility

	FFQ	3DR	Wilcoxon Signed Rank Test (p Value)	Correlation coefficient^2^	ICC^3^
	Mean ± SD	Mean ± SD			
Carbohydrate	262.22(92.28)	235.75(84.97)	0.013	0.7	0.52
Protein	63.42(22.62)	58.75(20.94)	0.059	0.63	0.54
Fat	64.02(25.76)	60.28(24.58)	0.136	0.61	0.51
Calcium	748.58(335.10)	696.48(272.37)	0.434	0.54	0.59
Fe	13.65(5.01)	12.77(4.78)	0.016	0.75	0.74
Magnesium	218.52(76.82)	201.07(81.82)	0.013	0.65	0.6
Vitamin C	97.48(54.55)	95.75(62.34)	0.211	0.77	0.62
Vitamin A	777.25(503.65)	745.43(532.87)	0.915	0.68	0.7
Vegetables	170.65(87.34)	175.79(107.75)	0.866	0.59	0.75
Fruits	280.38(155.49)	284.13(170.27)	0.135	0.68	0.65
Meat and its products	89.56(41.06)	78.41(34.25)	0.124	0.68	0.75
Dairy products	394.29(224.29)	370.29(192.16)	0.541	0.89	0.62

^1^ Wilcoxon signed-rank test were used to examine the difference between FFQ and 3DR with a significant p value < 0.001. ^2^Pearson or Spearman correlation coefficient were used to assess the correlation between variables with a p-value < 0.05 considered as significant. ^3^The intra-class coefficient for the comparison between FFQ1 and FFQ2. 3DR, dietary recall

## Discussion

The current study was conducted to evaluate the association between HEI and anthropometric status in primary school girls in Kerman. The results showed that children with higher HEI scores were more likely to be overweight than those with lower HEI scores. However, there were no significant associations between overall diet quality and other anthropometric indices. To the best of our knowledge, this is the first study to evaluate HEI in children aged 6–12 years old in Kerman.

Our study indicates a significant association between HEI score and the odds of being overweight. Similar findings were reported by Askari et al., who found that HEI was positively associated with the odds of being overweight in a cross-sectional study of 788 six-year-old Iranian children [6]. Also, in another cross-sectional study on Turkish adults, a positive association was found between HEI and BMI [18]. However, in contrast with our findings, Sundararajan et al. reported an inverse association between HEI and BMI in Canadian adults [19]. A systematic review indicated an inverse association between HEI and weight gain [20]. On the other hand, Azadbakht et al. found no significant association between HEI score and BMI of female students (n 265) aged 11–13 years [21]. Hurley et al. conducted a cross-sectional study and found no correlation between weight status in school-aged children and their adherence to the youth healthy eating index [22]. Moreover, Manios et al. demonstrated in a cohort study on 2,518 children aged 1 to 5 years that BMI status was not associated with HEI score [23].

The contradictory results might be attributed to variations in study design, populations, and dietary intake assessment tools. The age of participants significantly affects the results, as children in this particular age range have different eating habits and more control over their food choices. They might consume sugar-sweetened beverages, snacks, fast food, or other energy-dense food items related to weight gain but not considered in the HEI. The different eating habits in diverse populations should also be considered, especially since most diet quality assessment tools like Healthy Eating Index (HEI), Diet Quality Index (DQI), and Dietary Guidelines for Americans Adherence Index (DGAI) are designed for the US population.

Our research shows no significant association between the HEI score and anthropometric indices. Similar to our study, several studies did not show significant association between HEI score and anthropometric status [21–23]. However, some studies found a significant relationship between HEI scores and anthropometric indicators [18, 19, 24]. The lack of significant association in our study could be due to various reasons. Firstly, it could be due to the study’s cross-sectional design limited our ability to detect long-term energy imbalances. Secondly, the lack of association we observed may also be due to under or over-reporting of food intake by parents or guardians, who may need to be made aware of their child’s total food intake or changes in eating behavior. Lastly, the HEI scoring method treats all adequately consumed foods equally, even though different food groups may have varying impacts on anthropometric indices. For instance, processed foods and full-fat dairy are categorized as adequate components in the HEI but could contribute to increased weight status.

Our findings indicated that participants with higher HEI scores tend to have a higher intake of protein foods, fruits, vegetables, dairy, whole grains, and beans compared to participants with lower HEI scores. Previous studies indicates that consuming more whole grains, fruits, vegetables, and dairy products is associated with a decrease in anthropometric measurements [25, 26]. However, higher meat and beans intake have been associated with being overweight and obese [27, 28].

There are some strengths in the current study. First, the HEI-2015 was applied to investigate the association between diet quality and the odds of being overweight and obese in children in Iran for the first time. Second, a valid and reliable 185-item food frequency questionnaire was used in this study, which was specifically designed for participants that consumed local foods from Kerman City. Finally, the parents of the children were educated regarding healthy eating for eight weeks.

There are few limitations that should be described. The cross-sectional design of our study means that it cannot establish a definite cause-and-effect relationship. Also, it is critical to consider residual confounding variables that may impact the findings. Genetic diversity can serve as a potential source of residual confounding in epidemiological studies and is difficult to eliminate its effect entirely. While Iran has significant genetic diversity, Kerman has limited genetic heterogeneity. Finally, to address these few limitations, future studies should aim to investigate the relationship between HEI and anthropometric indices in primary school girls using a prospective design.

## Conclusions

We conclude that HEI-2015 had a significant association with being overweight among6-12 years old primary school girls in Kerman, while no significant association was observed between HEI and other anthropometric indices. We recommend implementing health education and intervention programs in primary schools in Kerman to promote a healthier eating index and reduce the risk of overweight and obesity among girls aged 6–12. Further research should be conducted on other age and population groups with different dietary patterns to understand the association between HEI and anthropometric indices.

## Data Availability

The datasets used and analyzed during the current study are available from the corresponding author on reasonable request.

## References

[1] Bogale TY, Bala ET, Tadesse M, Asamoah BO (2018). Prevalence and associated factors for stunting among 6–12 years old school age children from rural community of Humbo district, Southern Ethiopia. BMC Public Health.

[2] Organization WH. The double burden of malnutrition: policy brief Geneva: World Health Organization; 2017. WHO/NMH/NHD/17.3.[Google Scholar]; 2020.

[3] Mohammadi S, Bolourian M, Badpeyma M, Nasiri M, Alizadeh Ghamsari A, Movahedinia S (2022). Prevalence and risk factors of malnutrition among primary School children in Iran. Heal Provid.

[4] Neumann C, Harris DM, Rogers LM (2002). Contribution of animal source foods in improving diet quality and function in children in the developing world. Nutr Res.

[5] Motedayen M, Dousti M, Sayehmiri F, Pourmahmoudi AA (2019). An investigation of the prevalence and causes of malnutrition in Iran: a review article and meta-analysis. Clin Nutr Res.

[6] Askari M, Daneshzad E, Naghshi S, Bellissimo N, Suitor K, Azadbakht L (2021). Healthy eating index and anthropometric status in young children: a cross-sectional study. Clin Nutr ESPEN.

[7] Ohls TKENNEDYE, Carlson J, Fleming S (1995). The healthy eating index: design and applications. J Am Diet Assoc.

[8] You A. Dietary guidelines for Americans. US Dep Heal Hum Serv US Dep Agric. 2015;7.

[9] Krebs-Smith SM, Pannucci TE, Subar AF, Kirkpatrick SI, Lerman JL, Tooze JA (2018). Update of the healthy eating index: HEI-2015. J Acad Nutr Diet.

[10] Arzhang P, Abbasi SH, Sarsangi P, Malekahmadi M, Nikbaf-Shandiz M, Bellissimo N (2022). Prevalence of household food insecurity among a healthy Iranian population: a systematic review and meta-analysis. Front Nutr.

[11] Ranjit N, Macias S, Hoelscher D (2020). Factors related to poor diet quality in food insecure populations. Transl Behav Med.

[12] Seyedhamzeh S. The Conceptual Model of Food and Nutrition Security in Iran. 2017.

[13] Ahmadi E, Tehrani AR, Ahmadi A (2010). Prevalence of obesity, overweight and underweight among elementary school children in southern Iran, 2009. Am J Appl Sci.

[14] de Onis M, Onyango AW, Borghi E, Siyam A, Nishida C, Siekmann J (2007). Development of a WHO growth reference for school-aged children and adolescents. Bull World Health Organ.

[15] Garmaroudi GR, Moradi A (2010). Socio-economic status in Iran: a study of measurement index. Payesh (Health Monit.

[16] Hagströmer M, Oja P, Sjöström M (2006). The International Physical Activity Questionnaire (IPAQ): a study of concurrent and construct validity. Public Health Nutr.

[17] Kangani N, Mohammadi M, Zeinolabedin M, Bellissimo N, Azadbakht L (2022). Association between different types of edible oils and anthropometric indices Mood, and appetite among women. Int J Clin Pract.

[18] Koksal E, Karacil Ermumcu MS, Mortas H (2017). Description of the healthy eating indices-based diet quality in Turkish adults: a cross-sectional study. Environ Health Prev Med.

[19] Sundararajan K, Campbell MK, Choi Y-H, Sarma S (2014). The relationship between diet quality and adult obesity: evidence from Canada. J Am Coll Nutr.

[20] Asghari G, Mirmiran P, Yuzbashian E, Azizi F. A systematic review of diet quality indices in relation to obesity. Br J Nutr [Internet]. 2017/05/08. 2017;117(8):1055–65. Available from: https://www.cambridge.org/core/article/systematic-review-of-diet-quality-indices-in-relation-to-obesity/A58A7A614DDCB77A202A5C1183CEF988. 10.1017/S0007114517000915 28478768

[21] Azadbakht L, Akbari F, Esmaillzadeh A (2015). Diet quality among Iranian adolescents needs improvement. Public Health Nutr.

[22] Hurley KM, Oberlander SE, Merry BC, Wrobleski MM, Klassen AC, Black MM (2009). The healthy eating index and youth healthy eating index are unique, nonredundant measures of diet quality among low-income, African American adolescents. J Nutr.

[23] Manios Y, Kourlaba G, Kondaki K, Grammatikaki E, Birbilis M, Oikonomou E (2009). Diet quality of preschoolers in Greece based on the healthy eating index: the GENESIS study. J Am Diet Assoc.

[24] Nicklas TA, O’Neil CE, Fulgoni VL (2012). Diet quality is inversely related to cardiovascular risk factors in adults. J Nutr.

[25] McKeown NM, Meigs JB, Liu S, Wilson PWF, Jacques PF. Whole-grain intake is favorably associated with metabolic risk factors for type 2 diabetes and cardiovascular disease in the Framingham Offspring Study1234. Am J Clin Nutr [Internet]. 2002;76(2):390–8. Available from: https://www.sciencedirect.com/science/article/pii/S0002916523058975. 10.1093/ajcn/76.2.390 12145012

[26] Schulz M, Nöthlings U, Hoffmann K, Bergmann MM, Boeing H. Identification of a Food Pattern Characterized by High-Fiber and Low-Fat Food Choices Associated with Low Prospective Weight Change in the EPIC-Potsdam Cohort1. J Nutr [Internet]. 2005;135(5):1183–9. Available from: https://www.sciencedirect.com/science/article/pii/S0022316622102099. 10.1093/jn/135.5.1183 15867301

[27] Westerterp-Plantenga MS, Lemmens SG, Westerterp KR. Dietary protein– its role in satiety, energetics, weight loss and health. Br J Nutr [Internet]. 2012/08/01. 2012;108(S2):S105–12. Available from: https://www.cambridge.org/core/article/dietary-protein-its-role-in-satiety-energetics-weight-loss-and-health/CCA49F7254E34FF25FD08A78A05DECD7. 10.1017/S0007114512002589 23107521

[28] Azadbakht L, Mirmiran P, Esmaillzadeh A, Azizi T, Azizi F. Beneficial Effects of a Dietary Approaches to Stop Hypertension Eating Plan on Features of the Metabolic Syndrome. Diabetes Care [Internet]. 2005;28(12):2823–31. 10.2337/diacare.28.12.2823. 10.2337/diacare.28.12.2823 16306540

